# Process Parameter Optimization and Crack Formation Mechanism of Femtosecond Laser Welding of Fused Silica/6061 Aluminum Alloy

**DOI:** 10.3390/nano16181147

**Published:** 2026-09-14

**Authors:** Donghan Li, Yinzhi Fu, Jinlin Luo, Wen Li, Xianshi Jia, Kai Li, Lu Zhang, Yang Xiang, Cong Wang

**Affiliations:** 1 State Key Laboratory of Precision Manufacturing for Extreme Service Performance, College of Mechanical and Electrical Engineering, Central South University, Changsha 410083, China; 253711072@csu.edu.cn (D.L.); 243712090@csu.edu.cn (Y.F.); 233712115@csu.edu.cn (J.L.); 253711043@csu.edu.cn (W.L.);; 2 Wuhan Huaray Precision Laser Co., Ltd.—Professional Manufacturer of High-End Lasers, Wuhan 430074, China

**Keywords:** laser welding, fused silica, aluminum alloy, process parameters, fixture preload

## Abstract

Fused silica–aluminum alloy dissimilar connections are in urgent demand in fields such as aerospace optoelectronic packaging, vacuum optical windows, and micro-electro-mechanical systems, yet the dramatic mismatch in thermal-expansion coefficient and thermophysical properties between the two materials has long been a bottleneck for reliable joining. Current ultrafast laser welding of such heterogeneous systems still suffers from prominent problems, including stringent optical contact requirements, high crack sensitivity on the fused silica side, and unclear coupling mechanism between clamping conditions and joint defects. This work systematically studies the joining process of femtosecond laser welding of fused silica and 6061 aluminum alloy dissimilar materials, focusing on the effects of scanning speed, pulse energy, scanning spacing, and fixture preload on the shear strength, microstructure, and elemental diffusion behavior of the joints. The results confirm that scanning speed and scanning spacing have a synergistic effect on heat input density; the magnitude of the fixture preload is a key factor determining the interfacial residual stress and crack sensitivity. By optimizing the scanning speed (6 mm/s) and combining it with a low preload and 140 μm scanning spacing, a high-strength heterogeneous joint with uniform elemental transition and no macroscopic cracks can be obtained. This study provides a detailed process-optimization approach for high-quality laser welding of dissimilar brittle/ductile materials.

## 1. Introduction

With the rapid development of microelectronic packaging, optoelectronic devices, and aerospace fields, the demand for efficient joining of dissimilar materials, specifically fused silica and aluminum alloys, has become increasingly urgent [1,2,3]. Fused silica finds wide application in fields such as semiconductors, optical windows, and vacuum devices due to its excellent optical properties, high corrosion resistance, good wear resistance, and strong resistance to thermal shock [4]. Aluminum alloys, the third most abundant element in the Earth’s crust, are characterized by their superior electrical and thermal conductivity, resistance to atmospheric corrosion, easy to handle, and good plasticity; they are commonly used in industries including aerospace, transportation, electronic products [5], and Al-air batteries [6]. In aerospace optoelectronic pods and satellite payloads, 6061 aluminum frames are directly mated with fused silica optical windows to provide both load-bearing and thermal dissipation for precision optical systems [7,8]. In hermetic microelectronic packages and MEMS devices, 6063 aluminum housings are assembled with fused silica viewports to maintain internal cleanliness while enabling optical signal transmission [1,9]. For vacuum electronic and particle detection equipment, 5052 aluminum cavities are bonded with fused silica insulating windows, leveraging the low outgassing rate and vacuum compatibility of both materials [4,10]. Among these alloy systems, 6061 aluminum alloy is the most widely adopted research object in glass-to-metal ultrafast laser welding due to its balanced mechanical properties and widespread industrial use [8,11,12].

However, direct joining of these two materials remains challenging because of their substantial differences in thermophysical properties, especially their coefficients of thermal expansion. The large thermal-expansion mismatch can generate severe residual stresses during conventional brazing or diffusion bonding, resulting in brittle cracking of the fused silica [13,14,15]. Adhesive bonding and mechanical fastening can alleviate thermal-stress-related problems but often suffer from drawbacks such as outgassing, limited thermal stability, and insufficient hermeticity [16,17]. Ultrafast laser welding, characterized by highly localized energy deposition, a small heat-affected zone, high spatial precision, and non-contact processing, therefore provides a promising alternative for joining fused silica to aluminum alloys [11,12,18].

Irradiation of a material with a femtosecond laser beam first causes the incident energy to be absorbed by electrons; the process proceeds through three stages—electronic heating, electron-phonon coupling, and thermal diffusion or phase transition—and ultimately results in melting, vaporization, or plasma formation within the material [19,20,21]. The filamentation effect during long-focus propagation further regulates the spatial distribution of energy deposition and ablation efficiency, providing a flexible way to modulate the interfacial energy input [22]. However, this technology still faces challenges, including high sensitivity to assembly contact conditions, significant variability in joint strength, and difficulty in suppressing crack defects [7,23,24].

Under optical contact conditions, researchers have systematically investigated the welding mechanism and process optimization of fused silica–aluminum systems. Zhan et al. [12] studied femtosecond laser welding of fused silica to pure aluminum; using pump-probe microscopy, they identified multi-pulse-induced plasma splitting inside fused silica as a critical factor that dissipates laser energy and reduces interfacial energy deposition. By introducing a focal pre-compensation strategy to counteract self-focusing-induced focal shift, they improved the joint shear strength to 25.75 MPa, confirming that strict optical contact is a prerequisite for high-strength joints. At the atomic scale, molecular dynamics simulations have further revealed the microscopic melting and elemental interdiffusion behavior of the fused silica–aluminum interface under femtosecond laser irradiation, verifying the formation mechanism of the metallurgical transition layer from the atomic perspective [25]. On this basis, Li et al. [26,27] further adopted a femtosecond laser in burst pulse mode to carry out quantitative research on mirror-polished samples fixed by fixtures. They examined the influences of pulse energy, focal position, hatch spacing, and vortex beam shaping on joint performance, and combined finite element thermal–mechanical simulations to clarify the failure mechanisms. In terms of light sources, infrared femtosecond fiber lasers have also been applied to glass-metal welding, showing similar welding laws [28]. In addition to pulse mode modulation, spatial beam shaping strategies have also been adopted to regulate the thermal accumulation distribution and improve the welding quality in the thermal accumulation regime [29]. The influences of process parameters such as scanning speed and line spacing on joint strength have also been systematically verified in similar glass-aluminum alloy systems [30,31].

To break through the limitation that strict optical contact is incompatible with practical industrial assembly, researchers have developed non-optical-contact welding processes adaptable to large initial gaps. Various technical routes, including burst mode laser welding [32], non-contact welding of dissimilar material systems [33], and direct micro-welding and sealing of glass-ceramics [34], have been explored to expand the application scenarios of non-optical contact welding. Tan et al. [8] realized direct picosecond laser welding of aluminosilicate glass to 6061 aluminum alloy with an initial gap of 35 μm, requiring only simple ultrasonic cleaning of samples without additional polishing or preloading. The joint strength originates from the synergistic effect of Al–Si–O diffusion metallurgical bonding at the interface and mechanical interlocking provided by the glass modification zone, yielding a maximum shear strength of 15.98 MPa; they also found that arc-shaped microcracks in the glass matrix induced by thermal stress remain the primary failure source even when the interfacial mixing zone is free of defects.

Besides direct welding, interface modification and auxiliary methods have also been widely investigated to improve non-contact welding performance. Different surface treatment methods can significantly affect the interfacial bonding state and welding strength by regulating the surface morphology and oxide layer state of the aluminum alloy [35]. Solder paste-assisted burst laser welding can realize ultra-large molten pool formation, providing another idea for improving interface bonding [36]. As another technical route, Wang et al. [37] proposed a two-step method: a Cr_2_O_3_ interlayer was pre-deposited by millisecond laser, and then high-speed welding of fused silica and aluminum alloys was achieved under non-optical contact conditions. The formation of the ternary intermetallic compound (Si,Al)_2_Cr enables high-strength joints at a scanning speed up to 6000 mm/s with a shear strength of 20.7 MPa, offering a feasible solution for high-throughput manufacturing, though the introduced interlayer changes the chemical composition of the weld seam. In addition, relevant studies have explored pulse modulation strategies [38], industrial-grade process adaptation [39], and multi-type interlayer regulation schemes [10,40,41,42,43] to further expand the process boundary of glass-metal laser welding.

Overall, ultrafast laser welding of fused silica–aluminum alloy has achieved high joint strength under ideal optical contact conditions; however, stringent optical contact requirements limit its engineering applications. In terms of numerical simulation, thermal-fluid coupled models have been established for high-repetition-rate ultrafast laser welding of glass and metal, but the accurate quantitative simulation of the whole process is still restricted by the nonlinear absorption effect and self-focusing effect inside transparent media [44]. While the non-optical contact, large-gap welding process better aligns with real-world assembly practices, it exhibits a lower maximum joint performance limit and faces common challenges—such as the presence of microcracks on the glass side and significant strength variability at the joints. Previous works have carried out extensive research on parameter optimization [30,45,46] and interlayer composition control [9,47]. Regulating the spatial distribution of residual stress is recognized as the core approach to suppress crack formation and improve joint reliability, but the coupling mechanism between fixture preload and residual stress has not been systematically clarified [24]. Key unresolved issues in this field include interface reaction control, residual thermal stress mitigation, and the broadening of the process window. In terms of welding mechanism, previous pioneering studies have established the fundamental physical framework of ultrafast laser glass-to-metal welding: the femtosecond laser induces multiphoton ionization and thermal ionization at the interface through nonlinear absorption of transparent media, resulting in localized melting of the metal side and the formation of Al–Si–O chemical bonds accompanied by elemental interdiffusion [11,26,27]. However, existing mechanism research mostly focuses on the energy deposition and interfacial bonding behavior under ideal optical contact and sufficient clamping conditions. There is still a lack of systematic quantitative analysis on how process parameters and fixture preload jointly regulate the interfacial thermal stress, crack initiation, and final joint integrity. In particular, the bidirectional coupling mechanism between preload magnitude, elemental diffusion sufficiency, and residual stress level has not been clearly revealed, which restricts the process optimization and engineering application of this technology.

In this work, femtosecond laser welding of fused silica and 6061 aluminum alloy is systematically investigated under varied processing and clamping configurations. The effects of scanning speed, pulse energy, hatch spacing, and fixture preload on weld morphology, crack initiation, and mechanical properties are comparatively evaluated. Through optimization of processing parameters and interfacial contact conditions, crack-free fused silica–aluminum alloy joints with a shear strength of 7.02 MPa are obtained. Furthermore, the correlation among processing conditions, interfacial behavior, and crack formation is analyzed to elucidate the underlying mechanism of crack generation. These findings offer deeper insights into crack suppression and process window optimization for ultrafast laser welding of fused silica–aluminum dissimilar material systems.

## 2. Materials and Methods

The double-sided polished fused silica specimens used in this experiment had dimensions of 10 mm× 5 mm× 2 mm, whereas the 6061 aluminum alloy specimens had dimensions of 20 mm× 10 mm× 2 mm with single-sided polished. The physical properties of the two materials are shown in Table 1. Both the fused silica and 6061 aluminum alloy samples were cleaned using alcohol to remove surface contaminants before welding. The two specimens were clamped using a specially designed fixture, with the fused silica specimen positioned on top of the aluminum alloy specimen. The contact state between the materials was adjusted by varying the tension of the fixture. The high-preload condition corresponded to the maximum achievable clamping force, at which the fastening screws could no longer be further tightened. The low-preload condition was defined as the minimum preload required to produce visible Newton’s rings at the fused silica–aluminum alloy interface, indicating intimate interfacial contact. The focal position of the laser beam relative to the material interface was adjusted using a *Z*-axis translation stage.

**Figure 1:**
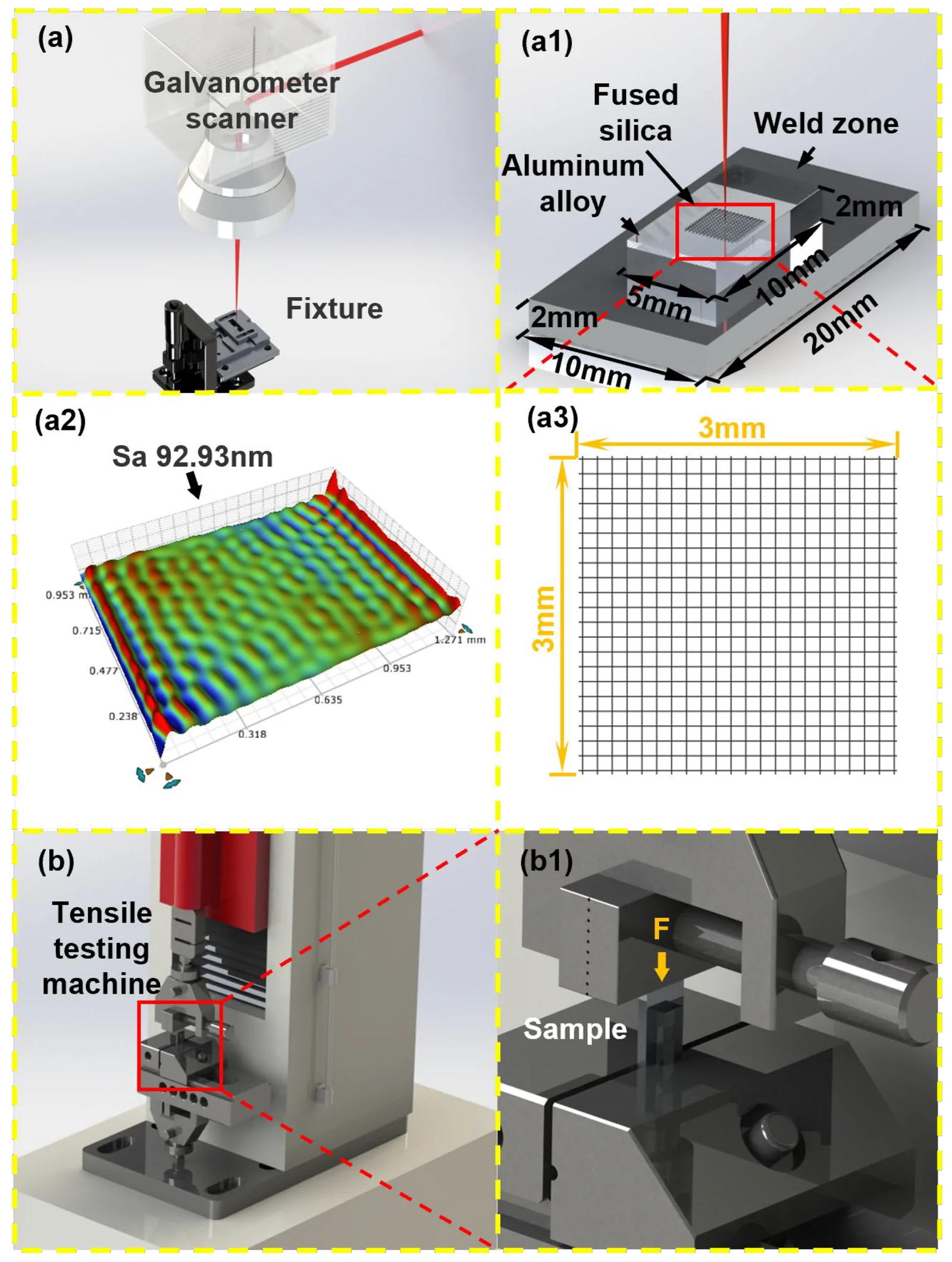
(**a**) Schematic diagram of the experimental setup; (**a1**) Schematic diagram of sample welding; (**a2**) Surface morphology and roughness of aluminum alloy measured by profilometer; (**a3**) Welding scanning path; (**b**) Schematic diagram of tensile-testing machine; (**b1**) Schematic diagram of sample positioning.

The experiment employed a femtosecond laser system integrated with a galvanometer scanner for welding (Figure 1a). The laser source (HR Platform-0203, Huaray Laser, Wuhan, China) has a wavelength of 1035 nm, a pulse duration of 200 fs, a focused spot diameter of 33.245 μm, and the galvanometer scanning system was equipped with an 80 mm focal-length lens. Both the laser parameters and the galvanometer scanning path were computer-controlled. The laser beam penetrated through the upper fused silica and focused at the interface, performing a grid-like scan within a 3 mm× 3 mm area to form the weld zone. The average surface roughness of the aluminum alloy is approximately 92.32 nm (Figure 1(a1–a3)). The repetition rate and defocus distance were fixed at 400 kHz and −150 μm, respectively. The investigated process parameters included scanning speed (5–10 mm/s), pulse energy (6.275–8.125 μJ), scanning spacing (100–160 μm), and two different levels of fixture preload (High/Low preload). The effects of these parameters on welding morphology and mechanical performance of the joints were systematically investigated. The shear strength of the joints was tested using a tensile-testing machine (Figure 1(b,b1)). The load was applied along the vertical direction until joint failure, and the maximum fracture load was recorded. In addition, Scanning Electron Microscopy (SEM) and Energy Dispersive Spectroscopy (EDS) were used for microscopic characterization of the joint fracture surfaces and the interfacial elemental transition zones.

## 3. Results

### 3.1. The Influence of Laser Parameters on the Quality of Welded Joints

#### 3.1.1. Scanning Speed

**Figure 2:**
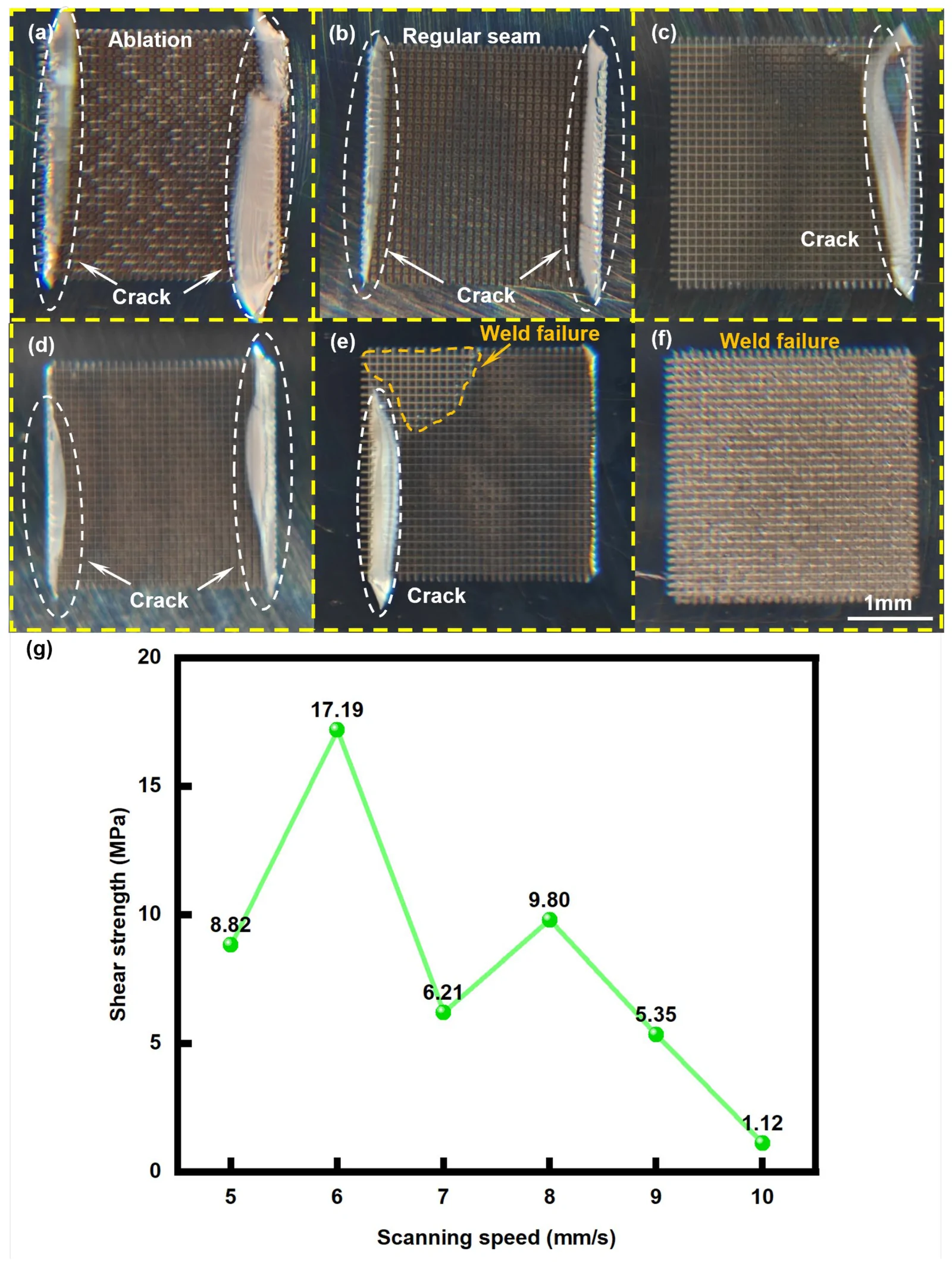
Welding macro-morphology at different scanning speeds: (**a**) 5 mm/s; (**b**) 6 mm/s; (**c**) 7 mm/s; (**d**) 8 mm/s; (**e**) 9 mm/s; (**f**) 10 mm/s; (**g**) Line plot of shear strength versus scanning speed.

The scanning speed directly determines the laser energy input per unit area and is a critical process parameter influencing joint formation and mechanical properties. Under the conditions of a repetition rate of 400 kHz, pulse energy of 6.275 μJ, defocus distance of −150 μm, scanning spacing of 100 μm, and high preload, a scanning speed range of 5–10 mm/s was selected. The macroscopic morphology of the weld seam and the shear strength of the joint at different scanning speeds are shown in Figure 2. At the speed of 5 mm/s, the laser energy input is excessive, leading to significant material ablation in the weld zone and the formation of cracks on the fused silica side. The weld seam exhibited regular geometry with no pronounced ablation or macroscopic cracks when the scanning speed increased to 6 mm/s, and the joint shear strength reached its peak. As the scanning speed increased further, the energy input per unit area decreased continuously, and the degree of melting and mixing of the interface materials diminished. Shear strength showed an overall declining trend when the scanning speed exceeded 8 mm/s. The energy input became severely insufficient at a scanning speed of 10 mm/s, resulting in weld failure and the shear strength approaching zero. An excessively low scanning speed, which leads to excessive energy input, can easily induce thermal cracking in the fused silica, whereas an excessively high scanning speed results in inadequate metallurgical bonding at the interface. Both scenarios degrade the mechanical properties of the joint. This variation trend is consistent with the parameter law widely observed in other glass-aluminum laser welding systems, where excessive energy input induces ablation and insufficient energy leads to weak bonding [30,31].

#### 3.1.2. Pulse Energy

**Figure 3:**
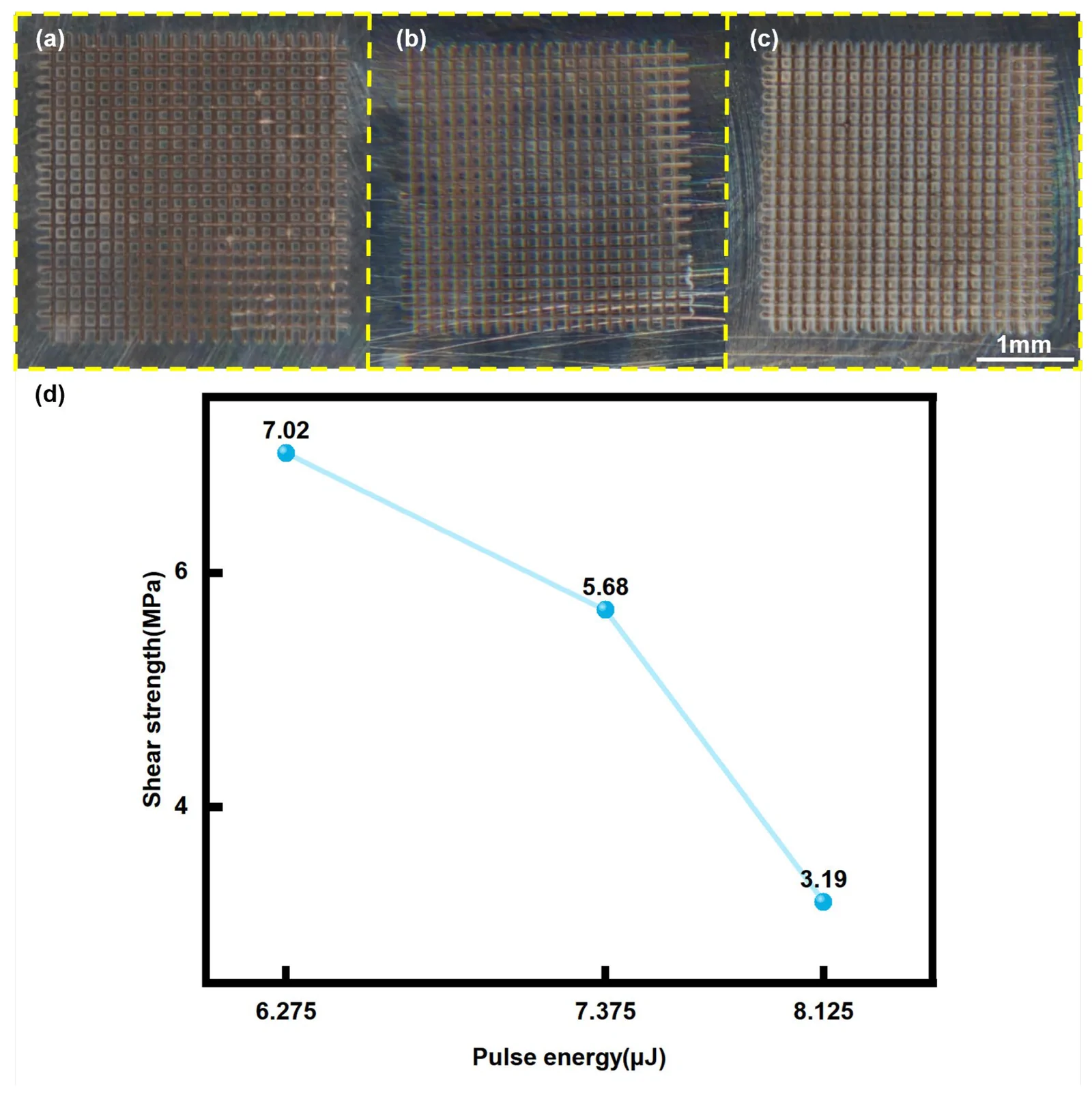
Welding macro-morphology under different pulse energies: (**a**) 6.275 μJ; (**b**) 7.375 μJ; (**c**) 8.125 μJ; (**d**) Line plot of shear strength versus pulse energy.

Under the conditions of a repetition rate of 400 kHz, scanning speed of 6 mm/s, defocus distance of −150 μm, and scanning spacing of 140 μm with low preload (Figure 3), the pulse energy increased from 6.275 μJ to 8.125 μJ. Under low-preload conditions, no obvious macroscopic cracks were observable around the weld joint; the shear strength continuously decreased from 7.02 MPa to 3.19 MPa. The self-focusing effect generated by the silica causes the focal point position to shift upwards when the pulse energy is too high, reducing the melting of the aluminum alloy, resulting in a decreased effective bonding area and a consequent reduction in connection strength. Therefore, controlling the appropriate pulse energy is a necessary prerequisite for ensuring joint quality.

#### 3.1.3. Scanning Spacing

**Figure 4:**
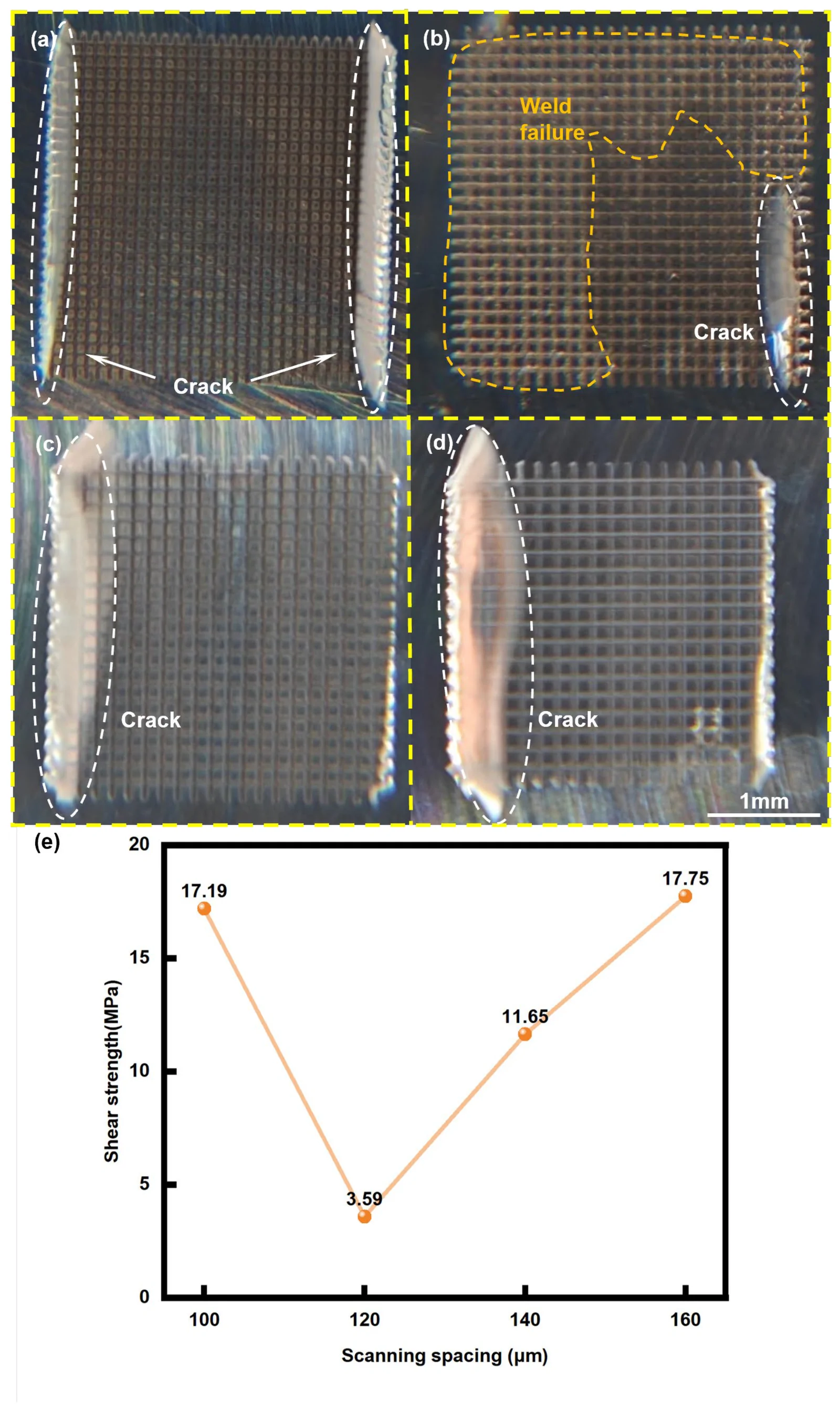
Welding macro-morphology at different scanning spacings: (**a**) 100 μm; (**b**) 120 μm; (**c**) 140 μm; (**d**) 160 μm; (**e**) Line plot of shear strength versus scanning spacing.

The influence of scanning spacing on shear strength exhibits a typical nonlinear fluctuation (Figure 4). Under the conditions of a repetition rate of 400 kHz, scanning speed of 6 mm/s, pulse energy of 6.275 μJ, defocus distance of −150 μm, and high preload, as the scanning spacing varies from 100 μm to 160 μm, the shear strength exhibited a distinctive V-shaped variation trend. At scanning spacings of 100 μm and 160 μm, the corresponding shear strengths reached 17.19 MPa and 17.75 MPa, respectively. Significant macroscopic cracks were observed around the weld seam. However, the shear strength dropped anomalously to 3.5 MPa when the spacing was 120 μm, associated with a large area of weld failure, due to minor fluctuations in the flatness of the aluminum alloy side, leading to poor fitting between the two materials during the welding process and finally resulting in a decrease in shear strength. This result indicates that the surface condition of the aluminum alloy material can affect the welding outcome.

### 3.2. Analysis of Interfacial Micro-Morphology and Elemental Diffusion Behavior

To further investigate the reason behind the V-shaped trend in shear strength at different scanning spacing, as well as the influence of varying fixture preload on crack formation, we examined the sample interfaces with scanning spacing ranging from 100 μm to 160 μm, and separately prepared welded samples with scanning spacing of 140 μm and 160 μm under low-preload conditions for comparison with samples welded under high-preload conditions.

Under the condition of high preload, variations in the scanning interval affect the thermal input density per unit area, thereby influencing the magnitude of interfacial residual stresses and the crack initiation behavior. We will investigate the influence of varying scanning spacing on shear strength by comparing the macroscopic and microscopic morphology as well as the elemental distribution of cross-sections obtained from samples with scanning spacings ranging from 100 μm to 160 μm.

**Figure 5:**
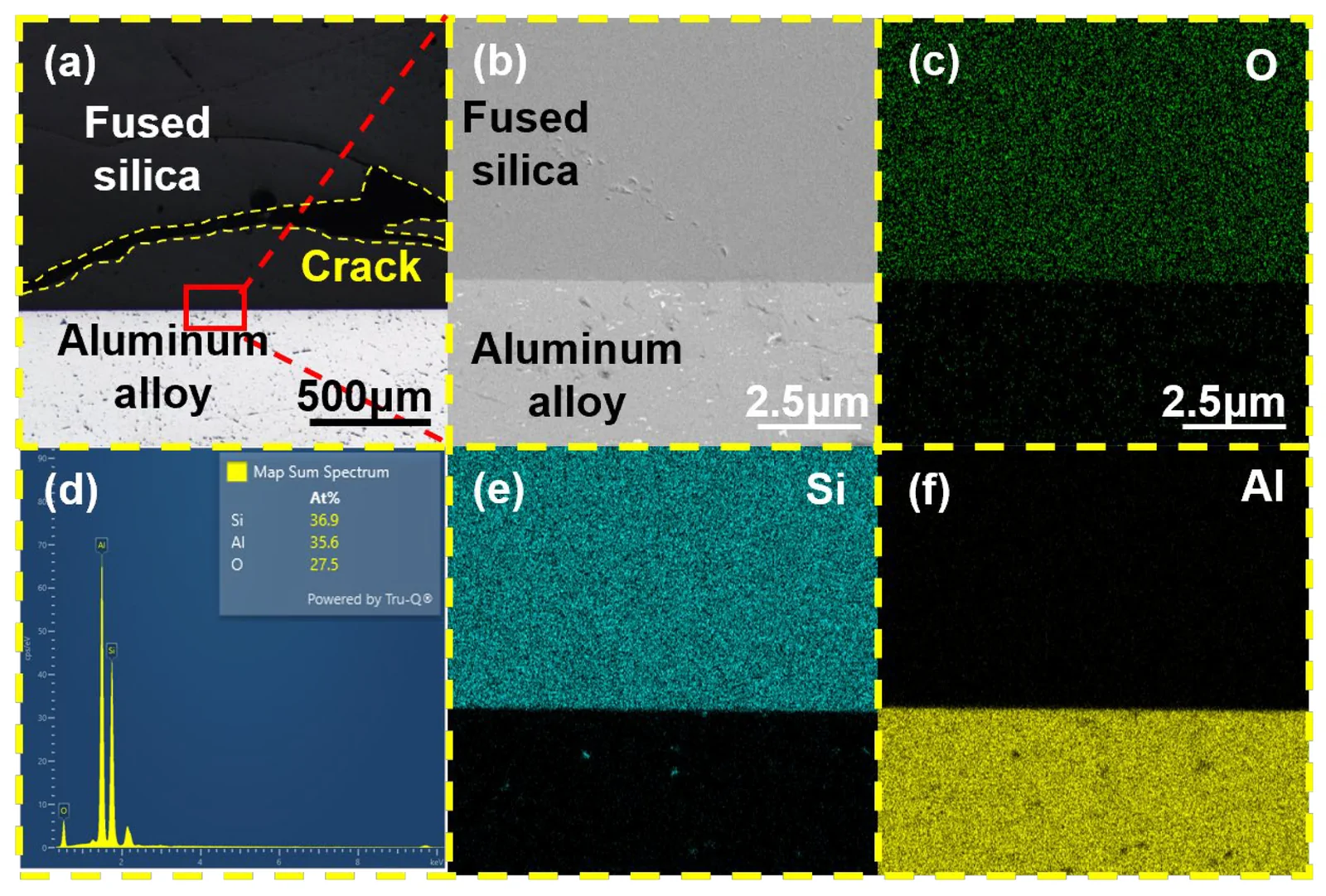
Scanning spacing of 100 μm under high preload. (**a**) Cross-section optical micrograph of the sample; (**b**) Cross-section SEM image of the sample; (**c**) Oxygen elemental distribution map; (**d**) EDS map-sum spectrum; (**e**) Silicon elemental distribution map; (**f**) Aluminum elemental distribution map.

As shown in Figure 5, when the scanning spacing is 100 μm, the low-magnification cross-sectional morphology (Figure 5a) shows obvious continuous penetrating cracks at the interface. The cracks originate from the fused silica side and propagate along the dissimilar bonding interface. The boundary between the two sides of the interface is clear, and the heat-affected zone is limited under higher magnification (Figure 5b). An excessively small scanning spacing leads to severe heat input accumulation per unit area. The huge CTE mismatch between fused silica and aluminum alloy induces extremely strong interfacial residual stress, which eventually triggers brittle cracking.

**Figure 6:**
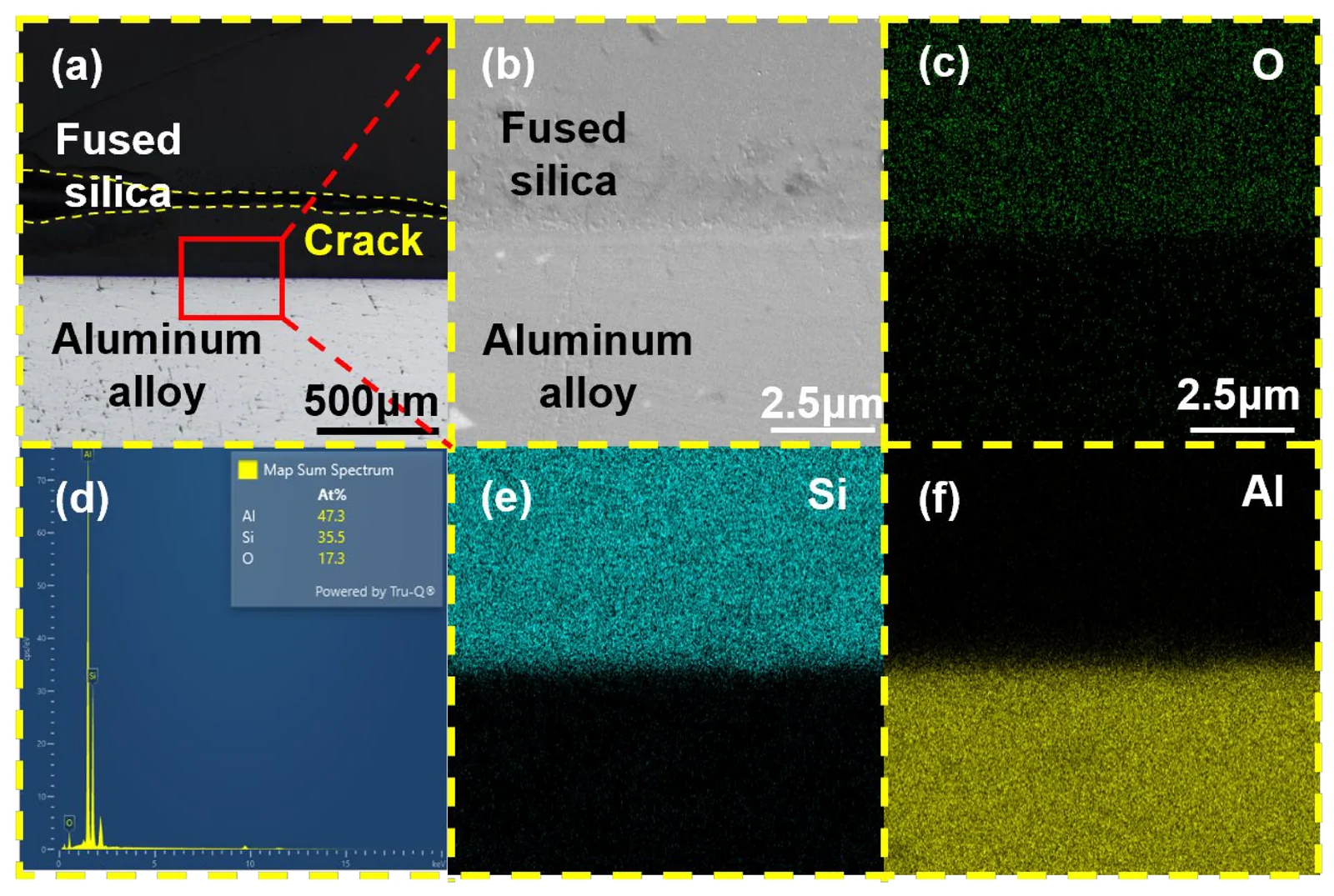
Scanning spacing of 120 μm under high preload. (**a**) Cross-section optical micrograph of the sample; (**b**) Cross-section SEM image of the sample; (**c**) Oxygen elemental distribution map; (**d**) EDS map-sum spectrum; (**e**) Silicon elemental distribution map; (**f**) Aluminum elemental distribution map.

Continuous macroscopic cracks still exist at the interface when the scanning spacing reaches 120 μm (Figure 6a), and the crack propagation scale shows no significant attenuation compared with the 100 μm condition. The heat input density at this spacing is still at a high level, and the thermal stress accumulation has not been effectively relieved, so the initiation of interfacial cracks cannot be suppressed. This further demonstrates that the sudden drop in intensity observed at a 120 μm spacing may be caused by surface quality variations on the aluminum alloy.

**Figure 7:**
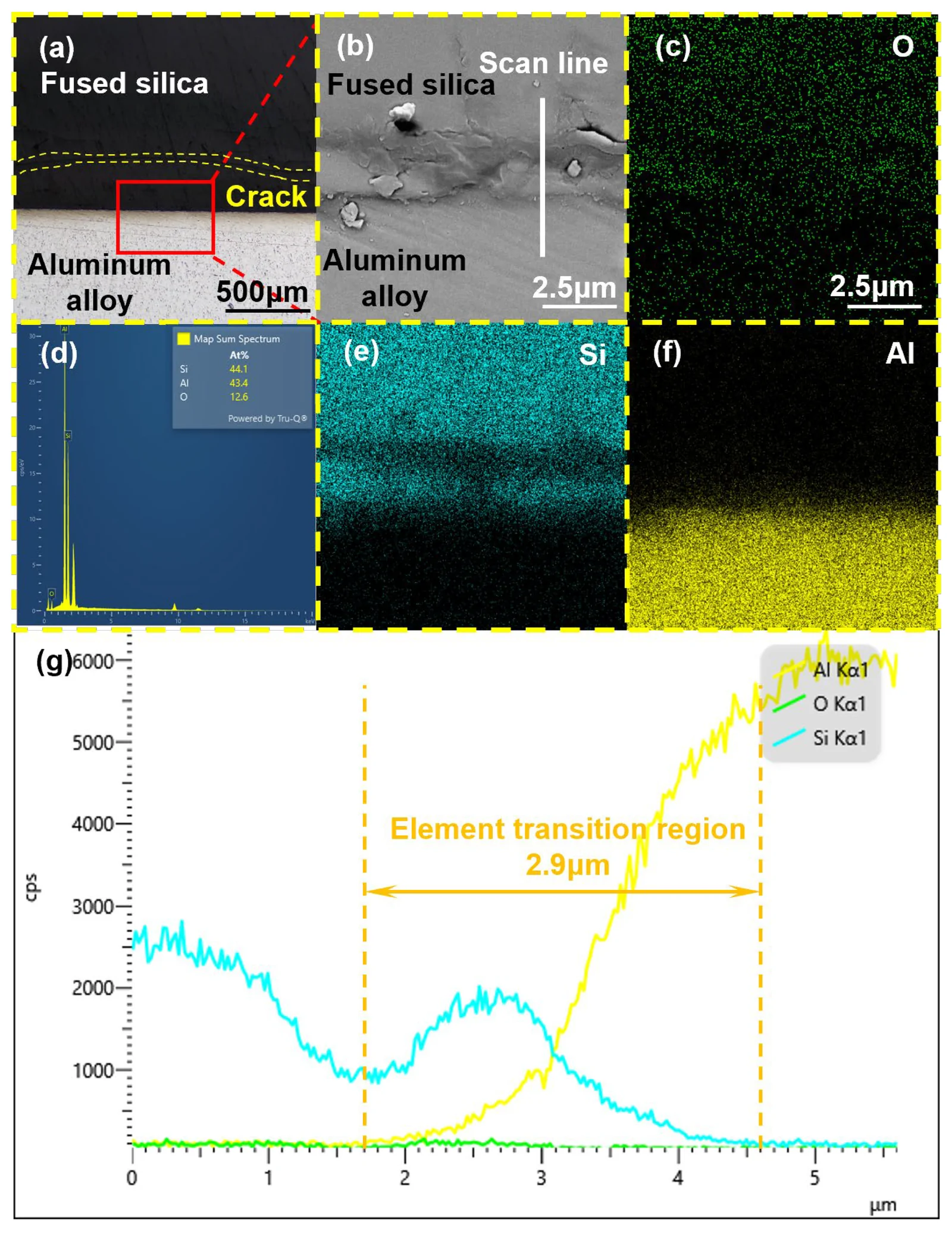
Scanning spacing of 140 μm under high preload. (**a**) Cross-section optical micrograph of the sample; (**b**) Cross-section SEM image of the sample; (**c**) Oxygen elemental distribution map; (**d**) EDS map-sum spectrum; (**e**) Silicon elemental distribution map; (**f**) Aluminum elemental distribution map; (**g**) EDS line-scan profile across the weld interface.

Obvious cracks can still be observed at the interface under high preload when the scanning spacing increases to 140 μm (Figure 7a). The laser scanning tracks are clearly distinguishable, and the cracks are distributed intermittently along the scanning path. Compared to the cases with interparticle spacing of 100 μm and 120 μm, cracks still exist. The observation in Figure 7c reveals a more extensive diffusion of oxygen, indicating that a chemical reaction may have occurred during the welding process. As shown in Figure 7g, the width of the element diffusion zone reaches 2.9 μm. Similar interfacial diffusion enhancement effects can also be achieved through filament-assisted double-sided laser welding, which verifies that sufficient interfacial contact and energy input are the prerequisites for adequate elemental interdiffusion [48].

**Figure 8:**
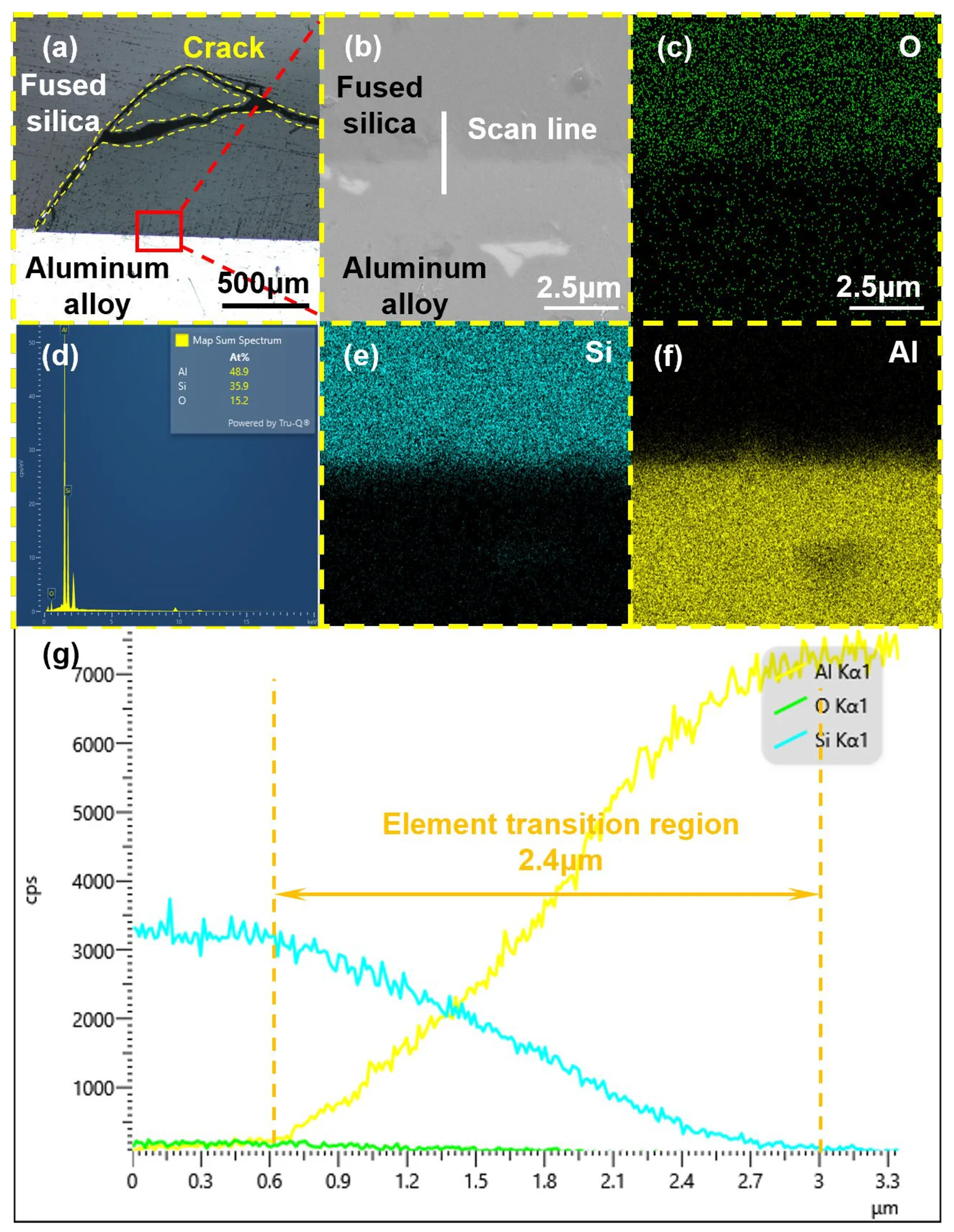
Scanning spacing of 160 μm under high preload. (**a**) Cross-section optical micrograph of the sample; (**b**) Cross-section SEM image of the sample; (**c**) Oxygen elemental distribution map; (**d**) EDS map-sum spectrum; (**e**) Silicon elemental distribution map; (**f**) Aluminum elemental distribution map; (**g**) EDS line-scan profile across the weld interface.

As shown in Figure 8, interfacial cracks still exist under high preload when the scanning spacing is 160 μm. This indicates that simply increasing the scanning spacing to reduce the overall thermal input density is insufficient to suppress the crack propagation length or crack width. Instead, crack formation is predominantly governed by the fixture preload, which dominates the bonding quality between the surfaces of the two materials. Figure 8c shows oxygen diffusion from the fused silica side toward the aluminum alloy side, although the width of the element diffusion region has decreased to 2.4 μm, which may be related to fluctuations in the surface condition of the aluminum alloy.

Overall, under the constraint of high preload, interfacial cracks cannot be completely avoided by optimizing the scanning spacing. Although the thermal input density decreases as the scanning spacing increases, it is difficult to prevent crack formation. In addition, the internal stresses generated in the sample during the welding process are dominated by the surface bonding state between the two materials.

### 3.3. Optimization of Fixture Preload Force for Crack Formation

#### 3.3.1. Sample Intensity Comparison and Cross-Sectional Analysis

An excessively small scanning interval leads to an increased deposited laser energy density; when there is a significant difference in the thermal-expansion coefficients of the two materials, residual thermal stresses are likely to arise, thereby increasing the risk of crack formation within the fused silica. Therefore, we focus our study on scanning intervals of 140 μm and 160 μm to compare the differences in interface morphology under high and low preloading conditions, which can help elucidate the core regulatory mechanism by which preloading influences crack formation.

**Figure 9:**
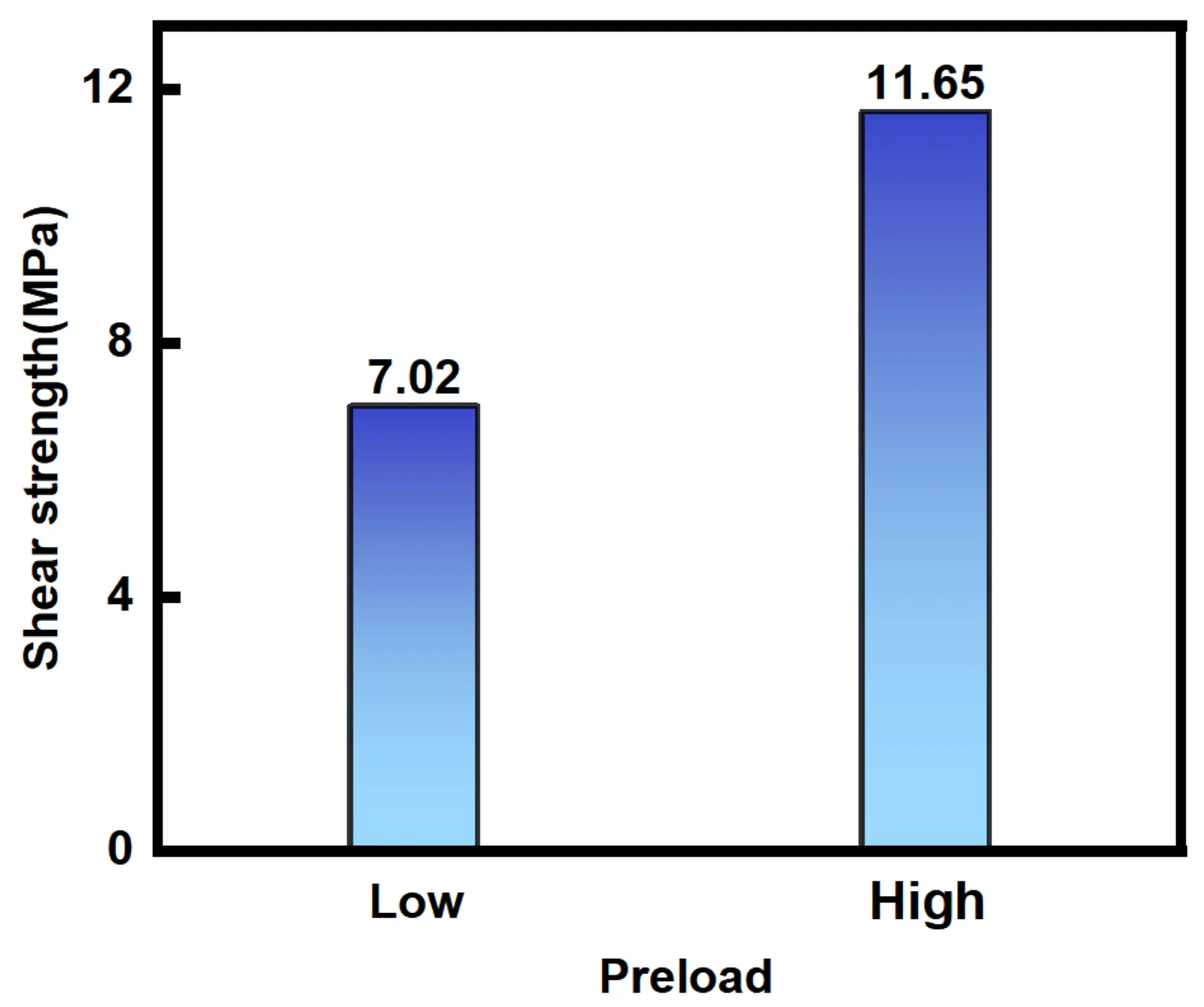
Comparison of shear strength of welded joints under different fixture preloads.

**Figure 10:**
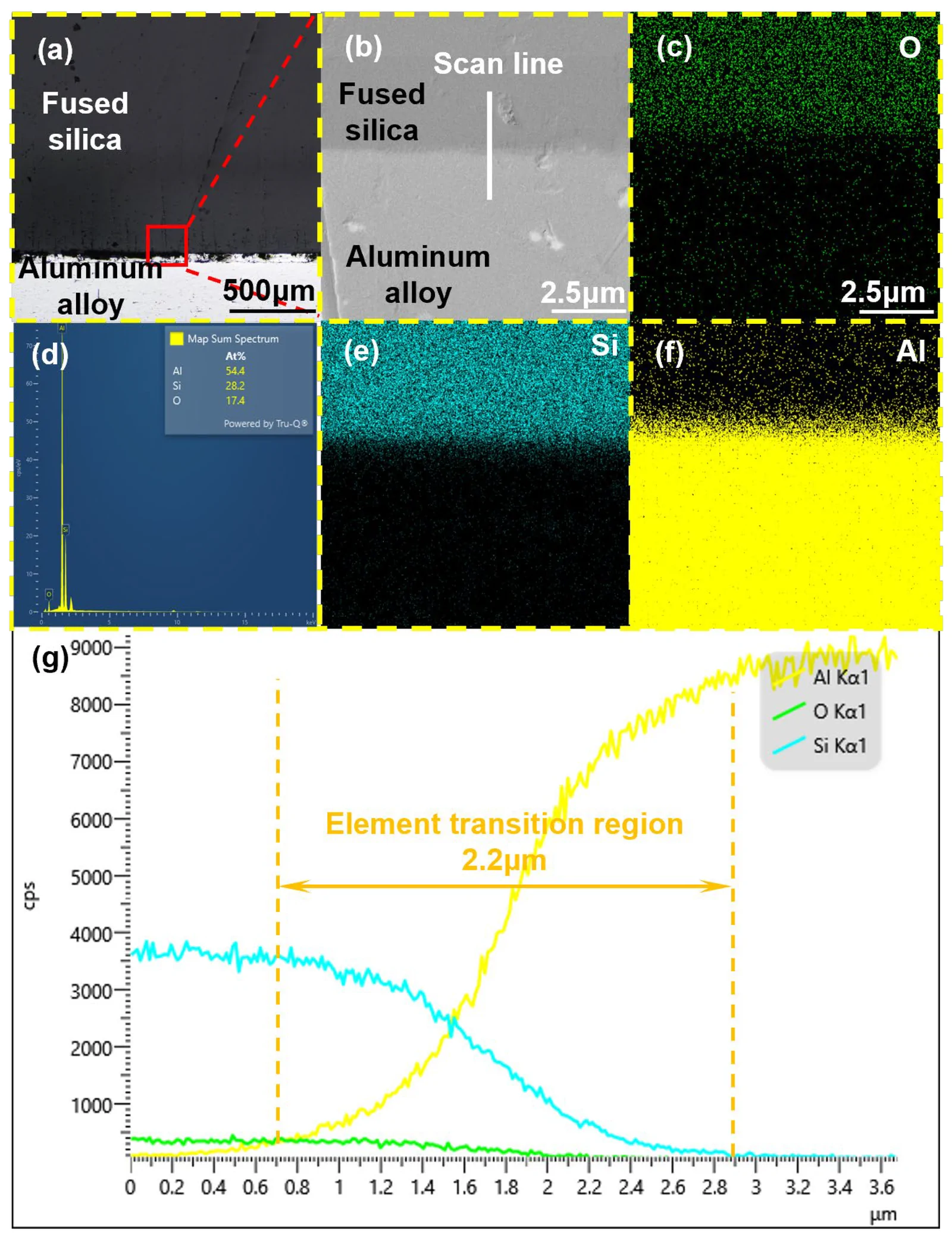
Scanning spacing of 140 μm under low preload. (**a**) Cross-section optical micrograph of the sample; (**b**) Cross-section SEM image of the sample; (**c**) Oxygen elemental distribution map; (**d**) EDS map-sum spectrum; (**e**) Silicon elemental distribution map; (**f**) Aluminum elemental distribution map; (**g**) EDS line-scan profile across the weld interface.

Figure 9 shows the shear strength of fused silica–aluminum alloy welded specimens under a scanning spacing of 140 μm, as a function of the different fixture preload forces. Under high-preload conditions, the specimen’s shear strength is higher, reaching 11.65 MPa; however, this is accompanied by obvious macroscopic cracks. Under low-preload conditions, crack formation can be prevented (Figure 10a), but the specimen’s shear strength is slightly reduced to 7.02 MPa. This indicates that controlling the degree of interfacial bonding in the specimen by adjusting the fixture preload force simultaneously affects both the shear strength and the occurrence of cracks.

**Figure 11:**
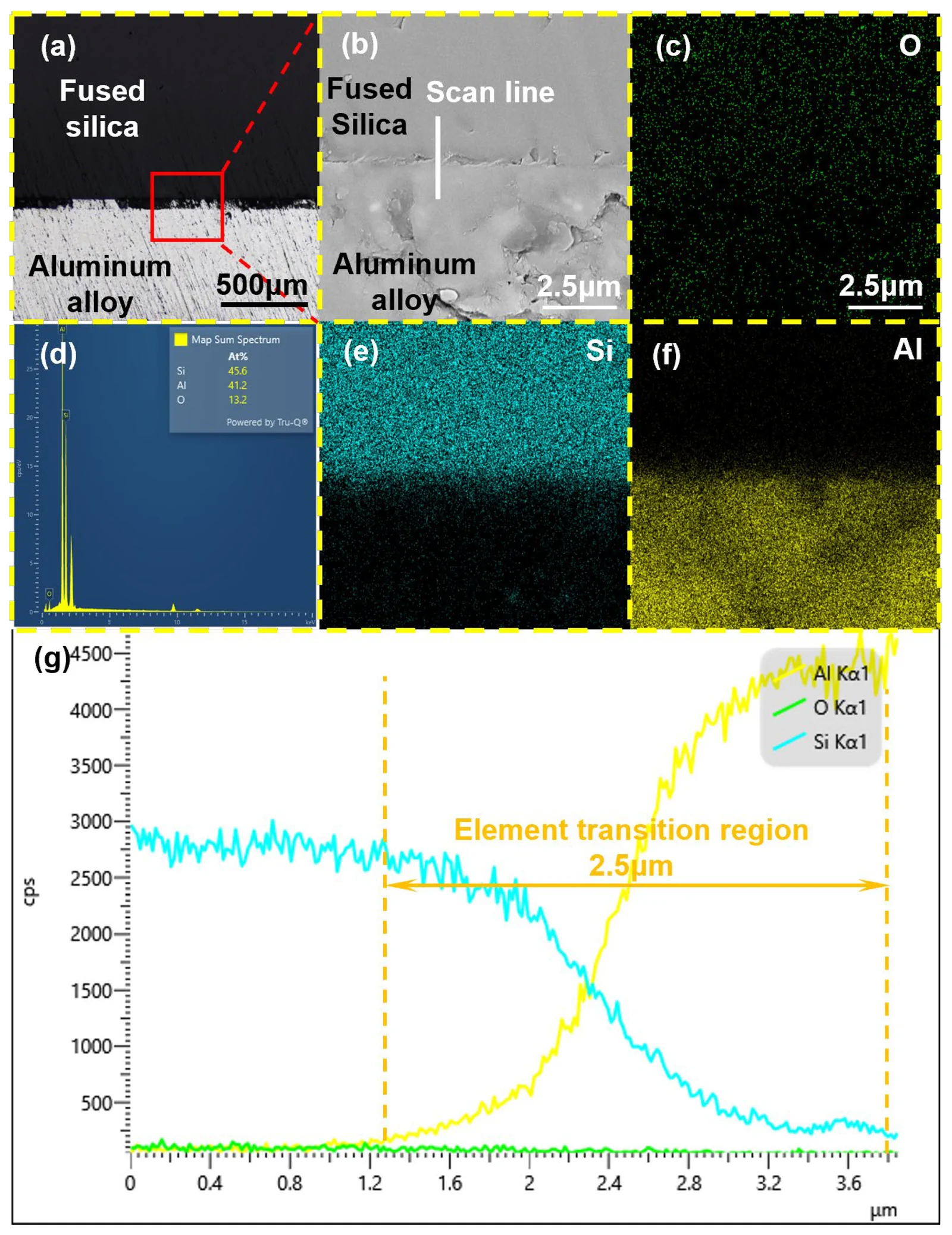
Scanning spacing of 160 μm under low preload. (**a**) Cross-section optical micrograph of the sample; (**b**) Cross-section SEM image of the sample; (**c**) Oxygen elemental distribution map; (**d**) EDS map-sum spectrum; (**e**) Silicon elemental distribution map; (**f**) Aluminum elemental distribution map; (**g**) EDS line-scan profile across the weld interface.

EDS line scan is a core quantitative method to characterize the degree of elemental diffusion at the interface of dissimilar materials. The experimental results correspond to Figure 7g, Figure 8g, Figure 10g, and Figure 11g. Taking the 140 μm scanning spacing as the core comparison group, the fixture preload directly regulates the sufficiency of elemental interdiffusion by changing the physical fit of the interface. The overall rule is that the elemental diffusion layer is thicker under high preload and thinner under low preload.

Under high-preload conditions, the large fixture preload enables tight physical contact at the fused silica–aluminum alloy interface, effectively eliminating assembly micro-gaps and providing sufficient diffusion channels and contact area for high-temperature atomic migration across the interface under femtosecond laser irradiation. The concentration curves of Si and Al elements form a wide cross-transition region at the interface with a relatively gentle concentration gradient, indicating that sufficient thermal-induced interdiffusion occurs at the interface and the metallurgical bonding layer is thicker.

At low preload, local micro-assembly gaps exist at the interface, and the two-phase materials cannot achieve completely tight contact. The effective contact area for atomic cross-interface diffusion is reduced, and the diffusion path is blocked. The concentration transition of Si and Al elements is steeper, and the width of the elemental transition zone is significantly narrower than that of the high preload sample. The interfacial interdiffusion degree is weakened, and the metallurgical reaction layer is thinner.

For the 160 μm scanning spacing group (Figure 8 and Figure 11), the difference in elemental transition zone width between high- and low-preload conditions is small. This may be due to certain fluctuations in the surface flatness of the aluminum alloy, which prevent tight interfacial fit of the two materials even under high preload. This result also indirectly indicates that interfacial fit is a prerequisite for determining the sufficiency of elemental diffusion: high preload eliminates gaps through forced compression, maximizes the atomic contact area at high temperature, thus promoting the cross-interface interdiffusion of Al and Si elements and forming a thicker metallurgical transition layer; the existence of interfacial gaps under low preload hinders atomic diffusion, and although the residual stress is lower, the physical conditions for elemental interdiffusion are weakened.

Notably, the diffusion layer thickness shows an inverse relationship with interfacial crack sensitivity: although high preload yields a thicker elemental diffusion layer and a higher metallurgical bonding degree, the high thermal residual stress induced by rigid constraints exceeds the brittle fracture threshold of the silica side, eventually inducing macroscopic cracks and instead reducing the actual load-bearing reliability of the joint. The diffusion layer thickness decreases under low preload, but the residual stress is effectively released, and the interface is free of crack defects, leading to better overall integrity and service stability of the joint.

#### 3.3.2. Fracture Surface Morphology and Failure Mode

**Figure 12:**
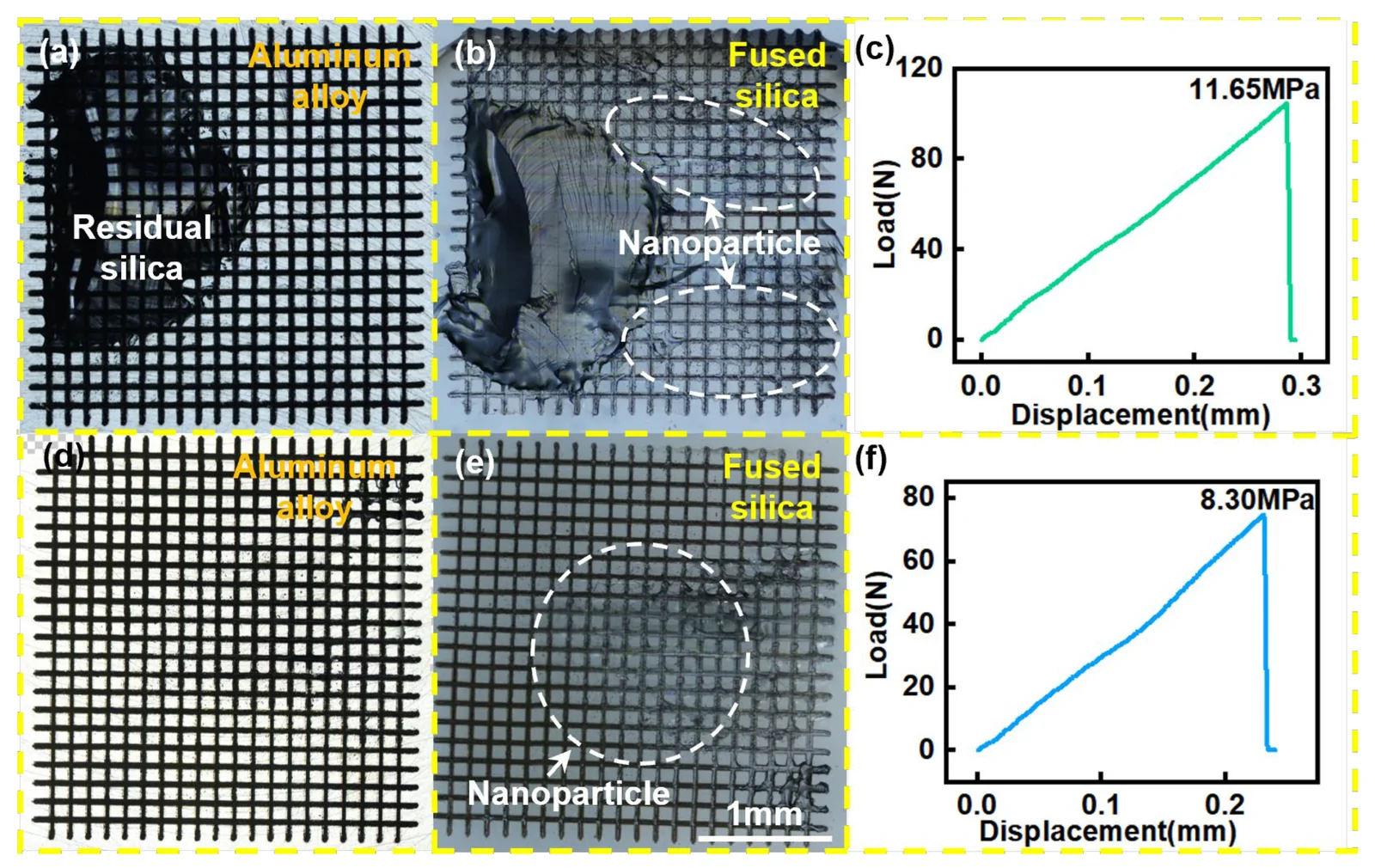
High-preload condition: (**a**) Fracture macro-morphology of aluminum alloy and (**b**) fused silica under 140 μm scanning spacing; (**c**) Load–displacement curve; Low-preload condition: (**d**) Fracture macro-morphology of aluminum alloy and (**e**) fused silica under 140 μm scanning spacing; (**f**) Load–displacement curve (Note: The value of 8.30 MPa represents the maximum strength; the average strength is 7.02 MPa).

At 140 μm scanning spacing, the fracture surfaces of the two preload levels correspond to different failure mechanisms:

For the high-preload fracture surface shown in Figure 12a,b, the fracture path propagates along the pre-existing cracks, showing typical brittle fracture characteristics with a flat and smooth fracture surface. The failure forms are mainly interfacial debonding and brittle spalling on the silica side.

Figure 12c shows the load–displacement curve under high-preload condition; its mechanical behavior and failure process can be divided into three distinct stages, which correspond to the interface morphology, crack characteristics, and fracture mode, respectively.

During the linear elastic deformation stage (displacement: 0–0.10 mm), the curve exhibits an approximately linear upward trend with a constant slope, corresponding to the elastic deformation phase of the interfacial bonding. In this stage, the load is entirely borne by the interfacial metallurgical bonding zone; the stress level is below the crack propagation threshold, and the pre-existing macroscopic crack has not yet undergone significant expansion. The deformation is primarily driven by the elastic deformation on the fused silica side and the elastic response of the interfacial bonding layer, demonstrating relatively high initial stiffness.

At the steady-state crack propagation stage (displacement: 0.10–0.25 mm), the curve slope gradually decreases, deviating from a linear relationship and marking the onset of the nonlinear deformation stage. For specimens subjected to high-preload forces, the pre-existing macroscopic cracks at the interface begin to propagate steadily along the joint surface under loading; simultaneously, a small amount of plastic deformation occurs on the aluminum alloy side. Together, these factors contribute to the continuous reduction in joint stiffness. The degree of nonlinearity in this phase directly reflects the propagation rate of the initial crack and confirms that the superposition of residual stresses and the external load under high-preload conditions accelerates crack initiation and propagation.

When it comes to the instability fracture stage (displacement: 0.25–0.35 mm), the load decreases rapidly after reaching its peak value (corresponding to a shear strength of 11.65 MPa); the load–displacement curve slopes steeply, without an obvious plastic extension plateau. When the crack extends to its critical length, instability propagation occurs, leading to an instantaneous brittle fracture of the joint, after which the load drops sharply to nearly zero. This behavior is fully consistent with the fracture surface morphology: under high pre-tension conditions, the fracture surface is smooth and flat, characterized primarily by brittle delamination on the fused silica side and interfacial cleavage along the crack; the fracture process absorbs little energy, representing a typical crack-controlled brittle failure mode.

Figure 12d,e show the low-preload fracture surface. There are more plastic deformation traces and material tearing features on the fracture surface, indicating higher interfacial bonding strength. More fracture energy is absorbed during the failure process, and the overall toughness of the joint is better than that under high preload. Figure 12c,f exhibit a similar trend; however, the peak shear strength in Figure 12f is slightly lower than that in Figure 12c. This is due to the reduced diffusion of both materials under low-preload conditions, which leads to a decrease in shear strength.

#### 3.3.3. Bidirectional Effect of Preload on Shear Strength

The comparison results of fixture preload and shear strength show that preload has a bidirectional regulation effect on the mechanical properties of joints:

The overall shear strength of joints under high preload is higher than that under low preload. The core reason is that high preload can eliminate sample assembly gaps, increase the effective contact area of the interface, and improve the proportion of physical fit and metallurgical bonding. However, the interfacial cracks accompanied by high preload will become natural fracture sources, greatly reducing the service reliability and impact resistance of the joints. Although the shear strength of joints decreases to some extent under low preload, the interface is free of crack defects. The failure mode of the joint transforms from brittle fracture along cracks to interfacial bonding failure, and the consistency and long-term stability of mechanical properties are more advantageous.

#### 3.3.4. Crack Formation Mechanism

Combined with the above experimental results, the regulation mechanism of fixture preload on crack formation in fused silica/aluminum alloy femtosecond laser welding can be summarized as follows.

**Figure 13:**
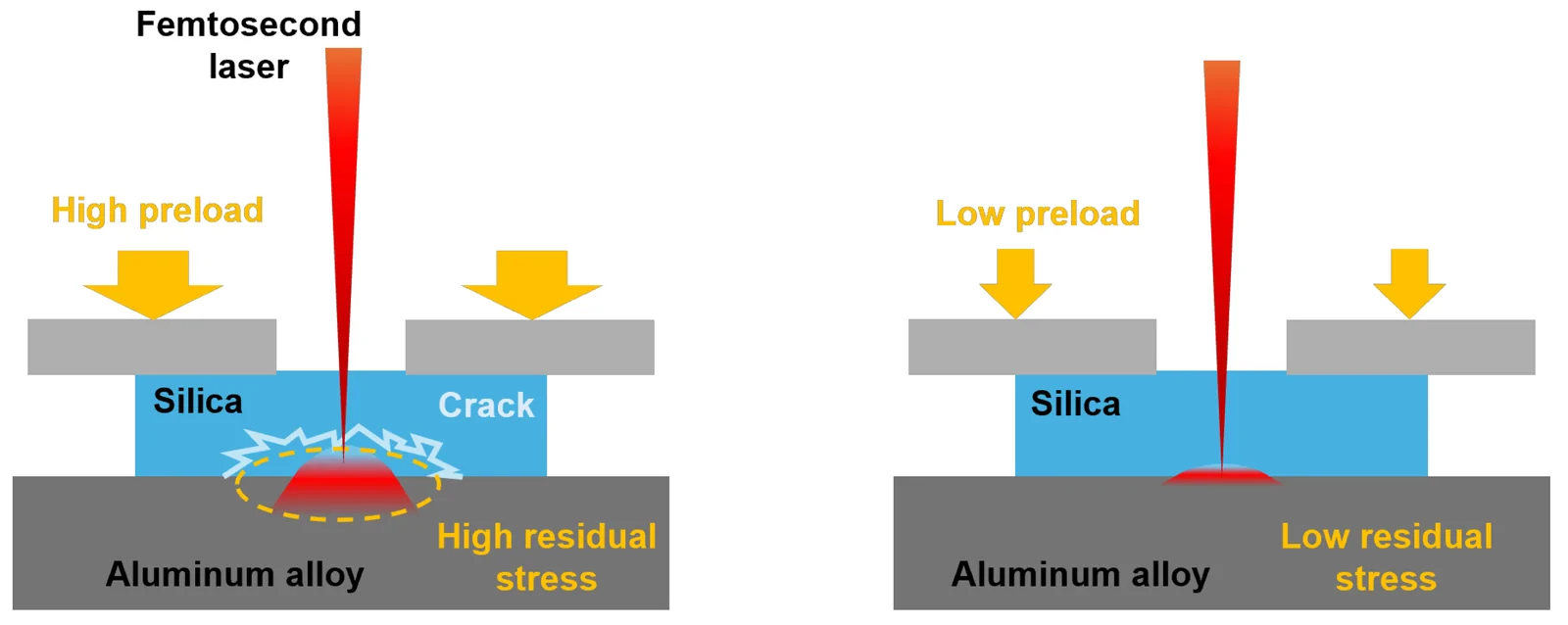
Schematic illustrating the effect of fixture preload on crack initiation.

As shown in Figure 13, after the femtosecond laser is focused on the dissimilar material interface, localized melting and interfacial Al–Si–O bonding occur following the classic nonlinear absorption and thermal diffusion mechanism. Due to the significant CTE mismatch between fused silica and aluminum alloy, severe thermal mismatch deformation is induced during the heating-cooling cycle. The magnitude of fixture preload essentially determines the constraint stiffness of the interface and the stress release path:

Under high preload, the interface is rigidly constrained. The aluminum alloy cannot release thermal stress through plastic deformation, and the residual stress is almost entirely borne by the brittle fused silica. When the peak thermal stress exceeds the fracture strength of fused silica, cracks initiate from the silica side and propagate along the interface, finally forming macroscopic penetrating cracks. Under appropriately reduced preload, the constraint stiffness decreases. The aluminum alloy can dissipate residual thermal stress through plastic yielding during the cooling process, which reduces the peak stress on the fused silica side below the fracture threshold, thus effectively suppressing crack initiation.

Appropriately reducing the fixture preload can provide plastic deformation space for the aluminum alloy during the welding thermal cycle, dissipate residual stress through deformation, and reduce the interfacial peak stress to effectively suppress cracks and obtain dissimilar material welded joints with no cracks and stable performance.

## 4. Conclusions

This work systematically investigates the control mechanisms governing the microstructure, mechanical properties, and crack formation at joints during femtosecond laser welding of fused silica/6061 aluminum alloy, focusing on the influence of process parameters and fixture preload. The scanning speed and scanning interval jointly determine the interface thermal input density: excessively low scanning speeds induce material ablation and thermal cracking, whereas excessively high speeds result in inadequate metallurgical bonding at the interface; increased pulse energy exacerbates the self-focusing effect within the fused silica substrate, thereby reducing the interface bonding strength; the shear strength exhibits a V-shaped variation dependence on the scanning interval.

The fixture preload exerts a bidirectional regulatory effect on joint performance: high preload eliminates interfacial gaps, promotes elemental interdiffusion, and enhances joint strength; however, the rigid constraint amplifies residual stresses arising from thermal mismatch, potentially inducing macroscopic brittle cracks on the fused silica side. Conversely, appropriately reducing the preload allows residual stresses to be released through the plastic yielding of the aluminum alloy, effectively suppressing crack propagation, with only a slight decrease in shear strength observed. By employing an optimized welding process featuring low fixture preload, a scanning speed of 6 mm/s, and a scanning interval of 140 μm, a welded joint free of macroscopic cracks, with uniform elemental distribution and a shear strength of 7.02 MPa can be obtained, ensuring superior structural integrity and service stability.

## Figures and Tables

**Table 1:** Physical properties of fused silica and 6061 aluminum alloy.

Materials	Density (g/cm^3^)	Melting/Softening Point (°C)	Thermal Conductivity (W/m·K)	Linear Thermal Expansion (K^−1^)	Specific Heat Capacity (J/kg·K)
Fused silica	2.20	~600	1.2–1.4	0.5–0.6 × 10^−6^	703–750
6061 Aluminum alloy	2.70	~585	151–202	2.32 × 10^−5^	897

## Data Availability

The original contributions presented in the study are included in the article; further inquiries can be directed to the corresponding author.
